# Annotations

**Published:** 1906-11-17

**Authors:** 


### ANNOTATIONS.

### Infants' Milk Depots.

The *British Journal of Children's Diseases* has recently sounded a note of warning against the dangerous influences that may be exercised by depôts established for the supply of milk for hand fed infants. These depôts are for the most part under the immediate direction of non-medical persons, and supply, according to a mechanical scale, milk adjusted to the supposed capacity of the infant as determined by its age. The milk supply of our large towns is not particularly free from contamination, and it is true the milk depôt may save the children of the poorer classes from this. There is, however, another side to the shield. The real evil exists in connection with the collection and distribution of the milk, and the reform which is radically necessary is the supervision and correction of these processes. To attempt to overcome the risks inherent in the present system by artificial methods in the milk depôt may be successful so far as it goes, but it leaves the origin of the mischief untouched. Again, it is not true that every infant of the same age needs milk containing one and the same percentage composition. Nor is it correct to say that increase of weight is a sure proof that the child is vigorous and thriving. Yet these are the ruling principles in the majority of milk depôts. There is truth and wisdom in the warning that professional patronage of milk depôts as controlled by "laymen, nurses, charity mongers, and other busy-bodies" will lead the public to believe that the mechanical rules of the depôt are sufficient, and that the question of infant-feeding is one in which neither professional guidance nor control is necessary.

### Ventilation of Operating Theatres.

A considerable number of medical men interested in the ventilation of operating rooms in hospitals assembled in the splendid new operating theatre of the Central London Hospital for Diseases of the Throat and Nose to hear Dr. Thomas Glover Lyon describe the system of heating and ventilating with warm air, with which his name has been associated. In this connection we would direct attention to our article on operating theatres on page 131. At the time of the demonstration the temperature of the operating theatre was equal to that of the external atmosphere—namely, 54°. Within a few minutes the incoming air, heated by these radiating electric lamps, raised the temperature to 61°, but a temperature of 65° can be produced without any sensation of that stuffiness which is met with in air heated by steam or water pipes. This theatre was difficult to heat on account of the marble walls, and so some hot-water apparatus has been added to be used on emergency or as a supplement when required. In this system the air never touches the surface of the heating apparatus. The heat is produced by the radiator and is swept away into screens at either side of the heat-generating apparatus, thus ensuring equable diffusion of the warm air. A stuffy room will cause the pulse-rate to rise fifteen in a minute, and the respiration about three—a very important point in connection with the administration of anæsthetics. No filters are employed. Cotton-wool filters soon become foul, and moist filters serve to moisten the air, which, in the English atmosphere, is not necessary. The total cost of working apart from installation with twenty radiators amounted to fourpence or fivepence an hour. Analyses of the air in this operating theatre have been made, and showed that the air was exceptionally free from pathogenic organisms, to such an extent as to make it appear that it had been sterilised by its passage over the radiators. The air supply is adequate to cope with the presence of from ten to twenty persons in the operating room.

### "Water for Domestic Purposes."

Inquiries have recently been made in various quarters as to what is meant in the Public Health Acts by "water for domestic purposes." A statement made in a recent issue of the *Local Government Board Chronicle* that this included a water supply for flushing closets seems to have surprised many persons. In many districts an extra charge is made for the water supply necessary for this purpose, and also for a bath. Such extra charges act, in many cases, as a deterrent to house-owners who would otherwise put these conveniences into their houses. Perhaps the prospect of the increased water-rate also acts as an inducement to tenants not to press their landlords to provide them. In either case the result may be regarded as bad for the health of the public. The same reason that operates against making people pay for the exact amount of water they consume—namely, that the economy thus induced would not be good for health—is valid against any extra charge being made for such necessary purposes as baths and the flushing of water-closets. One correspondent writes: "In this district an extra charge of 5s. per annum is made when a water-closet is put into a dwelling-house, and it acts as a deterrent in many cases, and privy middens are retained when water-closets would otherwise be used, to the sanitary advantage of the district." Yet it does not appear that this extra charge is legal. Where individuals have appealed against it the decisions of judges seem to have invariably been that the supply of water for both bath and water-closet are "domestic purposes" within the meaning of the Acts. In one case—that of Low *v.* the Lambeth Waterworks Company—it was held that this term includes "all the water that the occupiers of a house or a building in which they dwell use for the health, comfort, and enjoyment of themselves apart from any trade or business purpose." This definition would even seem to make it illegal to make an extra charge for the water used for watering a garden—other than a market-garden. Pleasure-grounds would also be exempted. Perhaps this would be straining the provisions of the Act too far, but, as a matter of public policy, it would be well were it universally understood that for such purposes as the flushing of a water-closet and the filling of a bath no extra charge could be made.

